# Evaluation of Tryptic Podocin Peptide in Urine Sediment Using LC-MS-MRM Method as a Potential Biomarker of Glomerular Injury in Dogs with Clinical Signs of Renal and Cardiac Disorders

**DOI:** 10.3390/molecules24173088

**Published:** 2019-08-26

**Authors:** Barbara Szczepankiewicz, Remigiusz Bąchor, Robert Pasławski, Natalia Siwińska, Urszula Pasławska, Andrzej Konieczny, Zbigniew Szewczuk

**Affiliations:** 1 Department of Internal Diseases with Clinic for Horses, Dogs and Cats, Faculty of Veterinary Medicine, Wroclaw University of Environmental and Life Sciences, Pl. Grunwaldzki 47, 50-366 Wrocław, Poland; 2 Faculty of Chemistry, University of Wroclaw, F. Joliot-Curie 14, 50-383 Wroclaw, Poland; 3 Veterinary Centre Nicolaus Copernicus University Toruń, Gagarina 7, 87-100 Toruń, Poland; 4 Department of Nephrology and Transplantation Medicine, Wroclaw Medical University, Borowska 213, 50-556 Wroclaw, Poland

**Keywords:** podocytes, podocyturia, mass spectrometry, liquid chromatography, myxomatous mitral valve disease, heart insufficiency, kidney disease, cardiorenal syndrome, dog

## Abstract

The early asymptomatic stage of glomerular injury is a diagnostic challenge in the course of renal and extra-renal disease, e.g., heart insufficiency. It was found that podocin, a podocyte-specific protein present in the urine, may serve as a biomarker in the diagnosis of glomerular disease in humans and animals including glomerulonephritis, glomerulosclerosis, amyloidosis, or nephropathy. Therefore, there is a need of development of the sensitive and straightforward method of urinary podocin identification. In this work, we report our extended research under the glomerular injury investigation in dogs by application of clinical examination and LC-MS-MRM method in the identification of canine podocin in urine samples. The LC-MS-MRM method is based on the identification of podocin tryptic peptide with the ^218^H-AAEILAATPAAVQLR-OH^232^ sequence. The model peptide was characterized by the highest ionization efficiency of all the proposed model podocin tryptic peptides in a canine urine sediment according to the LC-MS/MS analysis. The obtained results revealed the presence of the model peptide in 40.9% of dogs with MMVD (active glomerular injury secondary to heart disease = cardiorenal syndrome-CRS) and 33.3% dogs with chronic kidney disease. The potential applicability of the developed methodology in the analysis of podocin in canine urine sediments was confirmed.

## 1. Introduction 

Podocytes are glomerular epithelial cells that line the urinary side of the capillary loops. This highly specialized cells completely enwrap the glomerular capillaries with their primary and secondary processes. These processes combine with the neighboring foot processes forming a so-called slit diaphragm. Because of their anatomic localization at the outer aspect of the capillary loops, podocytes are constantly challenged not only by oxygen radicals, cytokines, immune complexes, and inflammatory processes but also by mechanical forces (i.e., glomerular hypertension and filtration pressure) [1,2,3,4]. Numerous scientific reports indicate that podocytes are useful in the diagnosis of glomerular disease in humans and animals such as glomerulonephritis, glomerulosclerosis, amyloidosis, familial glomerulopathy, and lupus nephropathy [5,6,7]. Moreover, podocyturia enables the differentiation between active and inactive processes [7]. It seems that the podocytes may be more useful in the diagnosis of kidney diseases in dogs than in cats. This is related to the specificity of the kidney disease in cats—primary glomerular disease was detected very rarely, since most cats with chronic kidney disease had tubulointerstitial pathologies [8,9,10]. In contrast to cats, most of dogs and humans suffer from glomerular injury [11]. 

Urinary podocytes are diagnosed based on the presence of associated proteins, such as podocin, nephrin, podocalyxin, synaptopodin, Wilms tumor protein-1, glomerular epithelial protein-1, and alpha actinin-However, to date, a podocyte-specific marker has not been identified. Podocin is one of the most robust biomarkers of podocytes [7]. It may be detected using immunological methods (immunohistochemistry, ELISA test), scanning electron microscope, or mass spectrometry.

In 2013 Garovic and co-workers [12] applied LC-MS-MRM method as a new tool in the investigation of podocyturia in urine sediments of preeclamptic patients. Briefly, the tryptic urine pellets hydrolysates were analyzed by LC-MRM-MS with addition of isotopically labeled internal standard. The podocin tryptic peptide with the ^39^H-QEAGPEPSGSGR-OH^50^ sequence was chosen as most promising for podocin identification and quantification in urine sediments, and the obtained results were in agreement with the immunological tests. Additionally, in 2014, the new approach for podocin quantification in the soluble fraction of urine, instead of urine sediments, were presented by Simon and co-workers [13]. They performed the assay of the endogenous podocin in urine from healthy donors and analyzed tryptic peptides in urine sample. In this study the new podocin tryptic peptide with the ^59^H-APAATVVDVDEVR-OH^71^ sequence was chosen for protein quantification by LC-MS-MRM. The presented application of LC-MS-MRM method in the investigation of podocin concerns the analysis of human urine samples. However, podocin may be a potent biomarker of some diseases also in animal urine samples including dogs, cats, or horses. Therefore, the development of animal urine sample preparation and determination of potent tryptic podocin fragment may allow the podocin identification in urine samples. Additionally, high sensitivity of LC-MS-MRM method may result in earlier diagnosis of glomerular pathologies in comparison to the commonly used methods. Very recently, we tested the applicability of LC-MS-MRM method in identification of podocin tryptic peptide in feline urine samples from animals with diagnosed chronic kidney disease (CKD), and the results obtained confirmed the utility of the developed method [14]. Our previous research on cats prompted us to further research on dogs because data from the literature indicate that podocytes are less useful in kidney disease diagnosis in cats compared to dogs. This is related to the species specificity of the kidney disease—primary glomerular disease was detected very seldom because most cats with chronic kidney disease had tubulointerstitial pathologies [9]. It is estimated that about 52% of dogs suffer from glomerular disease. Within this study, we focused on two stages of glomerular damage:

(1) Preclinical, asymptomatic, nonuremic stage. This model we achieved using dogs with symptomatic cardiac failure.
(2) Dogs with obvious kidney disease in uremic stage of kidney injury.

From a clinical point of view the most important information is: (i) is the sequence used in humans and cats useful in dogs. If not, (ii) whether the new sequence is useful, (iii) whether the LC-MS-MRM method is sensitive enough to detect glomerular damage not only in the symptomatic phase but also in the asymptomatic phase. In a previous article [14], asymptomatic cats were not included in the study. The main goal of this work was to investigate the applicability of LC-MS-MRM method in the analysis of podocin tryptic peptides in canine urine samples as a potent podocyte biomarker and to correlate the obtained results with the clinical, hematological, biochemical data and urine test results. 

## 2. Results 

**Figure 1:**
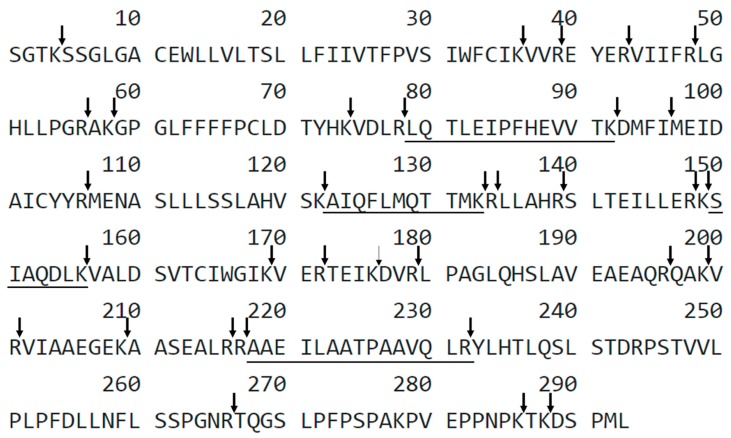
Sequence of canine podocin with marked trypsin cleavage sites determined according to the UniProt databases. The selected sequences were underlined. X-uncharacterized residue.

The aim of this work was to analyze the applicability of LC-MS-MRM method in the detection of podocin in the canine urine samples with diagnosed kidney dysfunction and to correlate the obtained results with the clinical, hematological, and biochemical data and with urine test results. The tryptic peptides with ^79^H-LQTLEIPFHEVVTK-OH^92^, ^123^H-AIQFLMQTTMK-OH^133 150^H-SIAQDLK-OH^156^, ^218^H-AAEILAATPAAVQLR-OH^232^ sequences were chosen as a potential podocin biomarkers (Figure 1). 

**Figure 2:**
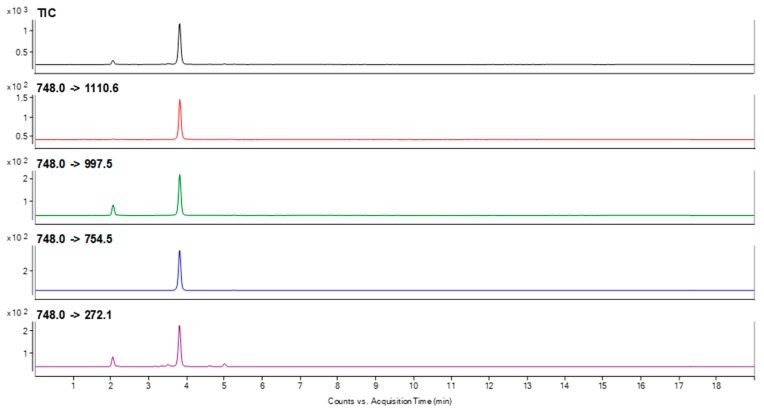
MRM chromatograms representing transitions corresponding to the model peptide with the ^218^H-AAEILAATPAAVQLR-OH^232^ sequence identified in the tryptic digest of a canine urine sediment sample from animal with diagnosed MMVD.

**Figure 3:**
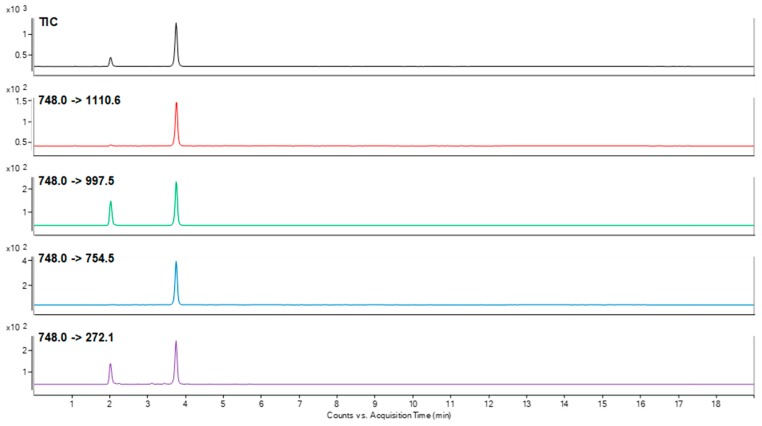
MRM chromatograms representing transitions corresponding to the model peptide with the ^218^H-AAEILAATPAAVQLR-OH^232^ sequence identified in the tryptic digest of a canine urine sediment sample from animal with diagnosed CKD.

To choose the best peptide for canine podocin identification that may be present in trace amount in urine samples, the sensitivity of detection for all of the obtained compounds was analyzed using LC-MS/MS and LC-MS-MRM methods. The obtained data clearly confirmed that peptide with the ^218^H-AAEILAATPAAVQLR-OH^232^ sequence was characterized at the subfemtomolar level of detection (2 × 10^−15^ mole). Other sequences were identified at the level of 10^−14^ mole. The performed experiment allowed to choose peptide with the ^218^H-AAEILAATPAAVQLR-OH^232^ sequence as a model for identification of podocine in canine urine samples. For the selected peptide, the MRM method was optimized using the [M + 2H]^2+^ (*m*/*z* 748.0) ion, as the most intensive signal on the obtained mass spectra. The following transitions 748.0→143.0 (b_2_), 748.0→997.5 (y_10_), 748.0→754.5 (y_7_), 748.0→1110.6 (y_11_), 748.0→272.1 (b_3_) were selected after manual development of the MRM method and used in the investigation of podocin in the canine urine sediment samples. The retention time of the chosen peptide under chromatographic separation conditions presented in materials and methods section was 3.8 min. The samples were prepared according to the procedure described in the materials and methods section. The obtained results for the sample obtained from the animals with diagnosed MMVD (active glomerular injury secondary to heart disease = cardiorenal syndrome-CRS) and CKD are presented in Figure 2 and Figure 3. 

The obtained MRM chromatograms for the analyzed urine sediment samples originating from animal with diagnosed MMVD (Figure 2) and CKD (Figure 3) show peaks corresponding to the transition characteristic for the selected canine tryptic podocin peptide with the ^218^H-AAEILAATPAAVQLR-OH^232^ sequence with the confirmed retention time. Based on the obtained MRM chromatograms, it can be assumed that podocin was found in the analyzed samples. 

The data obtained in the clinical and additional examinations for the analyzed dogs are presented in Table 1, Table 2, Table 3, Table 4, Table 5 and Table 6. 

Dogs in the heart and kidney groups were, statistically, significantly older than the healthy dogs. They have also significantly higher SAP (Table 1). There were no statistically significant differences in the WBC and HT between groups, and all the values were within the reference range. However, dogs in the control group had a significantly higher RBC than those in the kidney group. Dogs in the heart group had significant higher RBC than those in the kidney group. The heart group had a significantly higher concentration of HGB than the kidney group (Table 2).

All the dogs in the control group had normal values of urea, creatinine, Cyst C, and SDMA. The kidney group dogs with abnormal high urea and creatinine level had elevated, SDMA, Cyst C, and AspAT concentrations compared with control group. The heart group dogs had statistically lower iron concentrations in blood serum and lower Cyst C level than the healthy dogs. The levels of creatinine, SDMA, and chloride were statistically higher in the kidney group than in the heart group. Despite the average, level of aldosterone was visibly higher in heart and kidney groups vs. control; the large individual variations made the difference statistically not significant (Table 3).

The urine specific gravity as well as blood urine and UAC were significantly lower in the heart and kidney groups compared to the control group. Despite the average, level of detection of podocytes using LC-MS-MRM method was visibly higher in heart and kidney groups vs. control; the large individual variations made the difference statistically not significant (Table 4). LA/Ao and MR were higher in the heart group proving our hypotheses (Table 5).

Spearman’s correlation indicated that the increase in urine creatinine values strongly correlated with an increase in urinary podocytes in the control and kidney group, while no such association was observed in the heart group (Table 6). In the control group, a decrease in blood urea concentration was related to an increase in urine podocin. In the kidney group, an increase in blood urea and Cyst C was associated with an increase in urine podocin. 

## 3. Discussion

Here, we present our investigation on the application of LC-MS-MRM method in the analysis of podocin tryptic peptides in canine urine samples as a potent podocyte biomarker and correlation of the obtained results with the clinical, hematological, and biochemical data and urine test results.

Due to the fact that there are no commercial tests dedicated to the detection of canine podocin, it was necessary to first establish the best biological material for the study by testing urinary sediment and supernatant [15]. We found that urinary sediment subjected to low-speed, i.e., 2500× *g*, centrifugation is more useful for the diagnosis of podocin than the urinary supernatant. The low speed preserved the podocyte damage (the cell membranes) and the release of podocin into the supernatant. 

To analyze the possibility of application of LC-MS-MRM method in the investigation of podocin in the canine urine samples, the tryptic peptides sequences containing C-terminal arginine or lysine residue ^79^H-LQTLEIPFHEVVTK-OH^92^, ^123^H-AIQFLMQTTMK-OH^133 150^, H-SIAQDLK-OH^156^, ^218^H-AAEILAATPAAVQLR-OH^232^ were chosen, which may influence the ionization efficiency during LC-MS-MRM analysis. Finally, we focused our attention on a tryptic peptide with the ^218^H-AAEILAATPAAVQLR-OH^232^ sequence because it is located outside the transmembrane region and gives intensive signals during the MS/MS experiment corresponding to the b_2_, b_3_, y_7_, y_10_ and y_11_ fragment ions. Moreover the ^218^H-AAEILAATPAAVQLR-OH^232^ sequence meets the peptide selection criteria described by Mohammed and co-workers [16]. For the selected peptide the MRM method was optimized for the [M + 2H]^2+^ (*m*/*z* 748.0) ion, as the most intensive signal on the obtained mass spectra. In the investigation of podocin in the canine urine sediment samples the transitions 748.0→143.0 (b_2_), 748.0→997.5 (y_10_), 748.0→754.5 (y_7_), 748.0→1110.6 (y_11_), 748.0→272.1 (b_3_) were selected and used. Due to the fact that LC-MS-MRM is a sensitive method of protein detection, we observed the signals characteristic for the analyzed peptide on the MRM chromatograms not only in dogs with CKD but also in dogs with developing nephropathy such as MMVD.

A tryptic peptide with the ^218^H-AAEILAATPAAVQLR-OH^232^ sequence is different that was found in human and feline urine because the feline and human podocin tryptic peptide with the ^222^AVQFLVQTTMK^235^ sequence was used as model [14]. 

Using our methodology, the podocytes have not been found in the control group, which means no physiological podocyturia was found in healthy dogs. This finding shows differences between dogs and humans because in healthy humans the slow “physiologic” podocyturia (0.5 podocyte per mg creatinine in urine) has been reported [17]. Such podocyturia is higher in human urine of high concentration [17]. 

This article is the first confirming that urine examination using the LC-MS-MRM method allows the detection of glomerular damage prior to the occurrence of clinical signs, and enables the identification of the pathological processes occurring in the kidneys, without the need for a kidney biopsy. As studies have shown, a tryptic peptide with the ^218^H-AAEILAATPAAVQLR-OH^232^ sequence is different than this one found in human and feline urine because the feline and human podocin tryptic peptide with the ^222^H-AVQFLVQTTMK-OH^235^ sequence was used as model [14]. That is why it is simply impossible to transfer the results from humans and cats to dogs.

As a result, the analysis of the peptide with the ^218^H-AAEILAATPAAVQLR-OH^232^ sequence using LC-MS/MRM method is considered a primary non-invasive method enabling the differentiation of an active kidney disease from inactive damage. Painless, non-invasive sampling that does not require special animal preparation is a key advantage of this test. For this reason the proposed methodology may be in the future the method of choice in diagnosis of early stage of kidney insufficiency in dogs.

We found positive correlation in the kidney group of the amount of urine podocin with the increase in urine concentration. In the heart group, this connection does not reach the significance level, probably because in the heart group we observed the large individual variations in urine creatinine concentration. 

The podocin-positive cells detected in humans with glomerulonephritis included not only podocytes of the glomerular basement membrane but also parietal glomerular epithelial cells (PEC) and proximal tubule epithelial cells (PTEC) [7,18]. However, the majority of podocin positive cells were more podocytes than PEC and PTEC [7,18].

Another aim of this study was to determine whether the LC-MS-MRM method is sensitive enough to detect glomerular injury in the asymptomatic stage. Hence, a group of dogs with heart insufficiency was included in the study. The largest group of our study were dogs with symptomatic chronic heart failure. The dogs suffering the most common illness—myxomatous mitral valve disease—were chosen. We expected them to be at an increased risk of glomerular injury (CRS) because of the action of the renin-angiotensin-aldosterone system [19]. Previous studies found that podocytes contain cell surface receptors for angiotensin II [20], and that the activation of the renin-angiotensin-aldosterone system may cause podocyturia [21]. 

Hemodynamic changes may occur in dogs from the heart group and cause increased urine podocyte content (podocyturia). Using the LC-MS-MRM method, podocin was detected in diluted urine with a low specific gravity and a low urine creatinine concentration, which confirms the presence of CRS. 

We were unable to confirm glomerular injury in all the patients with heart failure. What suggests that 40.9% of these dogs are in active stage of glomerular pathologies and 59.1% in stable stage (without podocyturia). These results are of a great clinical importance because until now the frequency of CRS in dogs was estimated at 7.4–35.48% [22].

The performed study revealed the presence of the model podocin tryptic peptide in the analyzed canine urine sediments with confirmed active stage of glomerular damage. Therefore, it may be speculated that the proposed strategy of sample preparation and analysis may be useful in the investigation of podocin, biomarker of podocytes and podocyturia, in urine samples of dogs in the early (nonazotemic) stage of glomerular injury. Podocyturia should not be underestimated, and glomerular damage should be excluded in animals as podocytes are considered unable to proliferate after birth, limiting their regenerative potential [7]. The identification of patients with subclinical glomerular damage may be hampered due to the fact that podocyturia tends to be periodic.

Summarizing, we can say that liquid chromatography coupled with mass spectrometry (LC-MS) has become the method of choice in the analysis of protein mixture due to its sensitivity and accuracy, making the analysis of complex biological samples possible. Identification of compounds was based both on molecular masses of protein biomarkers and their fragments obtained in MS/MS or MRM mode. The major advantage of mass spectrometry in the protein biomarker investigation in comparison to the immunochemical methods is the possibility of analysis of many biomarkers in one experiment. Additionally, there is no need of antibody generation. Moreover, direct analysis of known compounds using MRM mode provides higher sensitivity and selectivity and allows fine-tuning of an instrument to search for the compound of interest and its fragment ions. This approach allows for the detection of low-abundance molecules in highly complex mixtures. Additionally, the targeted analysis, using MRM mass spectrometry, enhances the detection limit of peptides and allows continuous and fast monitoring of the specific ions of interest [23].

The clinical significance of the manuscript is selection of appropriate peptides for LC-MS-MRM method for the determination of podocytes in canine urine. The chosen podocin tryptic peptide with the ^218^H-AAEILAATPAAVQLR-OH^232^ sequences seems to be a promising biomarker to recognize the podocine. Using it we were able to confirm the active glomerular injury in 40.9% of dogs with heart failure (cardiorenal syndrome) and 33.3% of dogs with chronic kidney disease. In healthy dogs, there was no podocine positive samples. LC-MS-MRM method will be the first diagnostic toll enabling differentiation between the active and passive processes of glomerular injury. 

## 4. Materials and Methods

### 4.1. Ethics Approval and Consent to Participate

In accordance with the Experiments on Animals Act from 15 January 2015 (Journal of Laws of the Republic of Poland, 2015, item. 266), concerning the welfare of the animals used for research or teaching purposes, the provisions shall not apply to: (1) veterinary services as defined by the Act from 18 December 2003 concerning veterinary practices (Journal of Laws from 2004, No. 11, item 95 as amended in item 3), as well as agricultural activity, raising and breeding livestock according to the Animal Welfare Act, not designed to carry out medical procedures; (2) clinical veterinary studies carried out according to Article 37ah–37ak of the Act from 6 September 2001—Pharmaceutical Law (Journal of Laws from 2008, No. 45, item 271 as amended in item 4); (3) activity aimed at identifying animals; (4) capturing wild animals for biometric and systematic assessment; (5) veterinary procedures which to not cause pain, suffering, distress or permanent health impairment equal to or more invasive than the insertion of a needle. Hence, the study entitled “Investigation of tryptic podocin peptide in canine urine samples as a potential biomarker of glomerular injury using LC-MS/MRM method” does not require the approval of the Ethics Committee. All procedures were performed during the study with the owner consent.

### 4.2. Dogs

The study included 36 dogs that were patients of the Department of Internal Diseases with the Clinic of Horses, Dogs, and Cats, Faculty of Veterinary Medicine at the University of Environmental and Life Sciences in Wroclaw. Based on the anamnesis, clinical examination, and results of additional tests, the dogs were divided into three groups: (1) control group—eight healthy dogs, (2) heart group—twenty-two dogs, (3) kidney group—six dogs with CKD. 

There were two inclusion criteria in the control group: The absence of any signs of illness in the clinical examination (and in the last six months prior to the study) and normal results in all the laboratory tests. Dogs included in the heart group were in a C stable stage according to ACVIM classification [24]: Patients have a structural abnormality and current or previous clinical signs of heart failure caused by CVHD (chronic valvular heart disease, currently called myxomatous mitral valve disease (MMVD) [25]. Stage C includes all patients that have had an episode of clinical heart failure, despite improvement of their clinical signs with standard therapy (even if their clinical signs resolve completely). All the dogs had moreover hemodynamically significant mitral regurgitation, an enlarged left atrium and left ventricle (confirmed in an echocardiographic examination), and heart murmur. All the dogs were treated at home (chronic) with pimobendan 0.25–0.3 mg/kg POq 12 h, benazepril hydrochloride 0.25 mg/kg POq 24 h, spironolactone 2 mg/kg POq 24 h, and torasemide 0.1–0.6 mg/kg POq 24 h. Torasemide was used in relation to respiratory symptoms. The presence of abnormally high levels of serum creatinine, >1.4 mg/dL, 125 µmol/L, was an exclusion criterion.

Inclusion criteria to the kidney group based on the modified in 2017 IRIS staging of CKD [26]. All the dogs had renal azotemia lasting more than three months with the serum creatinine leave >1.4 mg/dL, 125 µmol/L. Prerenal and postrenal causes of azotemia were excluded. Dogs were classified as stage 2–4 according to IRIS classification and received renal diet and benazepril hydrochloride at a dose of 0.25 POq 24 mg/kg because of chronic albuminuria. 

The exclusion criteria for all the groups were body mass lower than 5.5 kg or higher than 22.0 kg, pregnancy, lactation, periods of growth and convalescence, dehydration, the presence of diseases that could significantly affect the blood flow in the kidneys (e.g., cancer, central nervous system diseases, the presence of acute inflammation process, food poisoning or an intake of medicines such as glucocorticosteroids, fluid therapy that may have significantly changed the kidney blood flow). Dogs with endocrine and immunological diseases and acute respiratory failure were also excluded from the study. All the procedures were performed in the morning. The examination was preceded by a 12-hour fasting period, and it was carried out without pharmacological or other restraint.

### 4.3. Clinical Examination with Measurements of Arterial Pressure

A history of previous diseases was obtained from the owner, and each dog was examined with particular emphasis on the circulatory and urinary systems. Systolic arterial blood pressure (SAP) was measured on the common digital artery after a 20 min rest, using model 811-B of the Doppler flow detector (Parks Medical Electronics Inc., Las Vegas, NV, USA). The pressure was calculated based on an average of 3-5 consecutive measurements.

### 4.4. Hematological and Biochemical Blood Test

Blood was collected from the v. saphena or v. cephalica antebrachii into 2 mL EDTA blood tubes. Blood collection was preceded by a 12-h fasting period. The hematological examination was performed immediately after the blood collection using an IDEXX X LaserCyte (Tokyo, Japan) hematology analyzer and a Horiba ABC animal blood counter (USA). The following parameters were measured: Concentration of hemoglobin (HGB), hematocrit (HT), red blood cell count (RBC), white blood cell count (WBC). The biochemical parameters were assessed in serum, after centrifugation using the Thermo Scientific Konelab Prime 30ISE (Vantaa, Finland) biochemical analyzer in the analytical laboratory of the Department of Internal Diseases of Horses, Dogs and Cats. The concentration of sodium (Na), potassium (K), ionized calcium (Ca2+), magnesium (Mg), iron (Fe), glucose, urea, creatinine, total protein, albumin, aspartate transaminase (AspAT), alanine transaminase (ALT), C-reactive protein (CRP) was also determined. The dogs were considered azotemic if the serum creatinine was >1.4 mg/dL, 125 µmol/L. The dogs were considered to have prerenal azotemia if they had increased serum urea concentrations without concomitant increased serum creatinine concentrations.

In addition, serum samples were transported to the IDEXX Ludwigsburg Germany Laboratories to determine concentrations of symmetric dimethylarginine (SDMA) and Cystatin C (Cyst C). SDMA was determined using an enzyme immunoassay dedicated to dogs. Cyst C was determined using liquid chromatography mass spectrometry—a nephelometric analysis dedicated to dogs. Reference values for urea, creatinine, Cyst C, SDMA, aldosteron that were used in this study have been published elsewhere [26,27,28,29].

### 4.5. Examination of Urine

The first morning urine was collected by the owner into a sterile container during spontaneous urination. The test was carried out immediately after receiving urine samples. The following parameters were evaluated during the physicochemical examination of urine: Colour, transparency, specific gravity, pH, protein, albumin, creatinine, glucose, blood, acetone and urobilinogen. The urine sediment was obtained through centrifugation for 10 min at 2500× *g*. Low speed spinning prevented podocyte damaged. Cell elements in the urine sediment were detect by a ZEISS Primotech microscope. The number of epithelial cells, blood cells, casts, and bacteria were assessed. A total of 0.7 mL of the urine sediment was stored at −80 °C until it was transferred to the Faculty of Chemistry, University of Wroclaw, Poland, in order to perform LC-MS-MRM. UPC was determined in the supernatant, and the UAC was calculated by dividing the protein albumin concentration (mg/dL) by the urine creatinine concentration (mg/dL). The result is a unitless ratio (dimensionless).

### 4.6. Echocardiography

A standard chest parasternal echocardiography was performed with a simultaneous ECG recording using a Hitachi Aloka F37 (Tokyo, Japan) echocardiograph with a 5–7.5 MHz sector probe. The aorta diameter (Ao), left atrium size (LA), end-diastolic (RVIDd) internal diameter of the right ventricle, end-diastolic (LVIDd) and end-systolic (LVIDs) internal diameter of the left ventricle, thickness of the interventricular septum at end diastole (IVSd) and end systole (IVSs), end-diastolic (LVFWd) and end-systolic (LVFWs) thickness of the free wall of the left ventricle were measured. The shortening fraction (FS) of the left ventricle was calculated using the Teicholz formula. Mitral regurgitation was measured using the continuous wave Doppler technic from the left parasternal two- or four-chamber view. The normal echocardiographic value of the size of left ventricle and left atrium was based on “Echocardiography: Principles of interpretation” [30]. 

### 4.7. Ultrasound Examination of the Abdomen

A standard abdominal ultrasound examination was carried out using the Hitachi Aloka F37 Japan machine with a 5–10 MHz micro convex and linear probe. Ultrasound examination allowed to exclude dogs with concurrent pathologies in the adrenal glands, spleen, liver, intestines, pancreas, reproductive truck, and lymphatics system. Additionally, the careful examination of urinary bladder and kidneys was performed to exclude neoplastic changes [31]. 

### 4.8. Chest X-ray Examination

Thoracic X-ray images were taken in right and left lateral recumbency and with a dorso-ventral projection using a Gierth HF200 digital camera (X-ray tube Toshiba, Tokyo, Japan). On radiographic assessment, the lung field, the dilation of the pulmonary artery and vein, the elevation of the distal part of the trachea towards the spine, and the presence of cardiomegaly were recorded (the size of the heart was assessed using the vertebral heart scale (VHS scale)) [32]. X-ray examinations were made to exclude patients with neoplastic or inflammatory changes and to confirm enlargement of the heart.

### 4.9. Peptide Synthesis—Reagents and Chemicals

All the solvents and reagents were used as supplied. Fmoc amino acid derivatives and the Fmoc-Arg(Pbf)-Wang resin (0.32 mmol/g), Fmoc-Lys(Mtt)-Wang resin (0.56 mmol/g) were purchased from Novabiochem. *N*-[(Dimethylamino)-1*H*-1,2,3-triazolo-[4,5-b]pyridin-1-ylmethylene]-*N*-methylmethanaminium hexafluorophosphate *N*-oxide (HATU) and trifluoroacetic acid (TFA) were obtained from IrisBiotech. Solvents for peptide synthesis (*N*,*N*-dimethylformamide (DMF), dichloromethane (DCM), and (*N*-ethyldiisopropylamine (DIEA) and tetraethylammonium bicarbonate (TEAB) were obtained from Sigma Aldrich; triisopropylsilane (TIS) was purchased from Fluka (Bucharest, Romania). Amicon^®^ Ultra Centrifugal Filters were purchased from Merck (Kenilworth, NJ, USA).

Model peptides on the Fmoc-Lys(Mtt)-Wang and Fmoc-Arg(Pbf)-Wang were synthesized manually in polypropylene syringe reactors (Intavis AG) equipped with polyethylene filters, according to a standard Fmoc (9-fluorenylmethoxycarbonyl) solid phase synthesis procedure.

### 4.10. Purification

The synthesized peptide was purified using the analytical HPLC Thermo Separation system and UV detection (210 nm) with a YMC-Pack RP C18 column (4.6 × 250 mm, 5 μm), and a gradient elution of 0–40% B in A (A = 0.1% TFA in water; B = 0.1% TFA in acetonitrile/H_2_O, 4:1) over 30 min (flow rate 1 mL/min). The main fraction, corresponding to the peptide, was collected, lyophilized, and confirmed by MS/MS experiment.

### 4.11. Mass Spectrometry

All ESI-MS experiments were performed on a micrOTOF-Q mass spectrometer (Bruker Daltonics, Bremen, Germany) equipped with standard ESI source. The instruments were used in the positive-ion mode and calibrated with the Tunemix™ mixture (Agilent Technologies, Palo Alto, CA, USA). The mass accuracy was higher than 5 ppm. The analyte solutions (70 μL) were introduced at a flow rate of 3 μL/min. The instrument parameters were as follows: The scan range of the micrOTOF-Q MS was 50–1600 *m*/*z*; nitrogen was used as the drying gas; the flow rate was 4.0 L/min, the temperature was 200 °C; the potential between the spray needle and the orifice was 4.2 kV.

### 4.12. CID

The singly ([M + H]^+^) and doubly protonated ([M + 2H]^2+^) precursor ions were selected on the quadrupole and subsequently fragmented in the hexapole collision cell. Argon was used as the collision gas. The obtained fragments were registered as an MS/MS (tandem mass spectrometry) spectrum. The collision energy (10–30 V) was optimized for the best fragmentation. Bruker Compass DataAnalysis 4.0 software was used for MS spectra analysis.

### 4.13. Urine Sample Preparation 

At the beginning of the study, we compared five samples of the urine sediment and supernatant from dogs with obvious and active glomerulonephritis to select the best material for testing. 

The urine sediment was stored up to a month at −80 °C and was thawed prior to analysis. After thawing, podocin was detected according to the following protocol. The collected urine sediment samples (0.7 mL each) were centrifuged for 10 min at 5000 rpm at 30 °C. The supernatant was discarded, and the pellet was re-suspended in 1 mL of 0.1 M TEAB buffer containing RapiGest™ SF at a concentration of 0.1% and sonicated for 5 min. The sample was transferred into the Amicon^®^ Ultra Centrifugal Filter and centrifuged at 4000 rpm for 15 min. The sample was then diluted with a 0.1 M TEAB solution and centrifuged again (washing out the low molecular weight impurities). In the next step, 100 µL of 0.2 M DTT was added, the sample was incubated for 1 h at 30 °C and centrifuged at 4000 rpm for 15 min. DTT was removed by washing the sample with a 0.1 M TEAB buffer and centrifugation. The groups were blocked by the reaction with 100 µL of 0.1 M CAM (1 h, room temperature). Excess of CAM was removed by washing the sample with 0.1 M TEAB and by centrifugation. The obtained supernatant was placed into an Eppendorf tube. Then, 50 μg of trypsin was added in 200 μL of 0.1 M TEAB, and the sample was incubated at 37 °C overnight. After digestion, 20 μL of formic acid was added, and the sample was lyophilized. The dry solid was dissolved in 5% MeCN/H_2_O and used for the LC-MS analysis. 

### 4.14. Liquid Chromatography-Mass Spectrometry (LC-MS) Analysis in Multiple Reaction Monitoring (MRM) Mode

To analyze the possibility of LC-MS-MRM method application of in the investigation of podocin tryptic peptide in the canine urine samples the tryptic peptides with the ^79^H-LQTLEIPFHEVVTK-OH^92^, ^123^H-AIQFLMQTTMK-OH^133^, ^150^H-SIAQDLK-OH^156^, ^218^H-AAEILAATPAAVQLR-OH^232^ sequences were chosen as a potential podocin biomarkers in canine urine sediments. The peptides containing C-terminal arginine or lysine residue which may influence the ionization efficiency during LC-MS-MRM analysis. Finally, we focused our attention on a tryptic peptide with the ^218^H-AAEILAATPAAVQLR-OH^232^ sequence (Figure 1) because it is located outside the transmembrane region, gives intensive signals during the MS/MS experiment corresponding to the b_2_, b_3_, y_7_, y_10_, and y_11_ fragment ions and meets the peptide selection criteria described by Mohammed and co-workers [16]. For all the synthesized peptides, the detection sensitivity was analyzed using LC-MS/MS and LC-MS-MRM method.

LC-MRM experiments were performed on an Agilent Technologies 6470 Triple Quad LC/MS apparatus, with an Agilent Technologies 1290 Infinity II system equipped with an Aeris Peptide XB-C18 column (50 mm × 2.1 mm) 3.6 μm bead diameter, equilibrated at 24 °C. The LC system was operated with a mobile phase, consisting of solvent A: 0.1% formic acid in H_2_O and solvent B: 0.1% formic acid in MeCN. The gradient conditions (B%) were from 5 to 80% B within 13 min. The flow rate was 0.3 mL/min, and the injection volume was 5 μL. The MRM method was developed manually, and the following transitions were chosen: 748.0→143.0 (b_2_), 748.0→997.5 (y_10_), 748.0→754.5 (y_7_), 748.0→1110.6 (y_11_), 748.0→272.1 (b_3_).

### 4.15. Statistical Analysis

Statistical analyses were performed using StatSoft Statistica PL 12.0 Software. Data were expressed as mean and standard deviation (±SD) or median (dependent of distribution of variables). The normal distribution of data was analyzed using the Shapiro-Wilk test. One-way ANOVA with the Fisher’s LSD post-hoc test was used to compare normally distributed variables. The Kruskal-Wallis test was used for non-normally distributed or non-parametric variables. A simple or multiple ordinal regression test was used to describe the relationships between variables. A *p* value < 0.05 was considered significant. 

The research does not require the consent of the ethics committee because the experimental procedures did not go beyond the framework of clinical diagnostics.

## 5. Conclusions

We demonstrated a new methodology for the detection of podocytes in canine urine samples based on the identification of a podocin tryptic peptide with the ^218^H-AAEILAATPAAVQLR-OH^232^ sequence using LC-MS-MRM method. The presented strategy allows us to identify the presence of podocin in canine urine. We found that urinary sediment is more useful for the diagnosis of podocin than the urinary supernatant.

LC-MS-MRM method is an exceedingly sensitive method, allowing the distinction of active kidney injury as it detects podocin in animals with glomerular injury (CRS and MMVD). Diagnostics of podocyturia using LC-MS-MRM method is a very promising method, which can greatly facilitate the unambiguous identification of podocin, could improve the diagnostic of early stage of glomerular injury, distinguish the active stage from inactive, and facilitate monitoring of patients’ treatment to avoid kidney damage during therapy. 

LC-MS-MRM method confirmed podocyturia in 40.9% of dogs with MMVD and 33.3% dogs with CKD. In accordance with our assumptions, podocin was not found in the urine samples of healthy dogs. 

## 6. Limitation

The limitation of our study was the detection of a single podocin marker and a relatively small number of dogs. In dogs, this marker does not differentiate podocytes, PEC, and PTEC. On the other hand, all these cells are present in active glomerulonephritis and should not be detected in healthy animals. It is likely that different podocyte populations (depending on the dominant protein) were diagnosed at various stages of the disease. Hence, the use of a selection of different proteins targeting podocytes would increase the sensitivity of the method. Podocyte-specific proteins should be located in different cell compartments: In the glycocalyx, the cytoplasm, and on the basal side of the podocytes. 

## Figures and Tables

**Table 1:** Clinical data of dogs in the control group, heart group (dogs with mitral regurgitation ACVIM (American College of Veterinary Internal Medicine) Class Cc), and kidney group (azotemic dogs). Data are presented as average ± standard deviation with the exception of body weight, presented as median ± quartile.

Characteristics	Control Group	Heart Group	Kidney Group	*p* Value
Number of dogs (female/male)	N = 7 (5/2)	N = 22 (9/13)	N = 6 (1/5)	
Age (years)	5.93 ± 3.01	10.91 ± 2.24	11.33 ± 2.58	<0.001 *^
Body weight (kg)	13.63 ± 6.63	9.55 ± 4.84	8.50 ± 5.63	NS
SAP	134.38 ± 6.20	161.60 ± 29.70	165.00 ± 33.90	0.034 *^

SAP: systolic arterial pressure. * Statistically significant differences between control group and kidney group, ^ statistically significant differences between healthy and heart group, NS: statistically nonsignificant.

**Table 2:** Hematological results of dogs from the control group, heart group (dogs with mitral regurgitation ACVIM Class Cc), and kidney group (azotemic dogs). Average ± standard deviation.

Variables	Control Group	Heart Group	Kidney Group	*p* Value
RBC T/L	6.93 ± 0.71	7.11 ± 0.77	5.60 ± 1.07	0.002 *#
HGB mmol/L	9.98 ± 1.17	16.66 ± 24.60	8.23 ± 1.27	0.008 #
HT %	48.44 ± 4.99	48.27 ± 5.62	42.67 ± 9.07	NS
WBC G/L	8.20 ± 2.81	9.14 ± 3.01	9.43 ± 5.65	NS

RBC: red blood cell count, HGB: concentration of hemoglobin, HT: hematocrit, WBC: white blood cell count. * Statistically significant differences between the control group and kidney group, # statistically significant differences between the heart and kidney group, NS: statistically nonsignificant.

**Table 3:** Blood biochemistry of healthy dogs (control group), dogs with mitral regurgitation ACVIM Class Cc (heart group), and the kidney group (azotemic dogs). Average ± standard deviation.

Variables	Control Group	Heart Group	Kidney Group	*p* Value
Urea mmol/L	5.20 ± 1.54	9.63 ± 10.51	23.08 ± 15.63	0.012 *
Creatinine µmol/L	105.88 ± 44.90	85.59 ± 27.50	295.83 ± 140.35	0.001 *#
SDMA µg/dL	10.75 ± 1.39	14.10 ± 5.40	25.70 ± 5.70	0.001 *#
Cyst C mg/L	0.93 ± 0.14	1.50 ± 0.60	2.28 ± 1.15	0.002 *^
Total protein g/L	59.00 ± 6.07	63.10 ± 4.34	58.50 ± 8.14	NS
Albumin g/L	30.88 ± 2.47	32.64 ± 3.26	28.98 ± 5.32	NS
Mg mmol/L	0.78 ± 0.10	0.79 ± 0.13	0.88 ± 0.09	NS
Na mmol/L	143.55 ± 1.91	145.66 ± 2.79	145.08 ± 4.25	NS
K mmol/L	4.57 ± 0.33	4.72 ± 0.47	4.74 ± 0.31	NS
Cl mmol/L	110.16 ± 0.64	108.30 ± 3.37	114.12 ± 4.76	0.004 #
Ca^2+^ mmol/L	1.31 ± 0.06	1.29 ± 0.07	1.34 ± 0.26	NS
Fe µmol/L	32.64 ± 7.27	24.42 ± 5.99	28.84 ± 1.29	0.048 ^
CRP mg/L	1.74 ± 0.39	2.93 ± 1.72	2.08 ± 0.37	NS
Glucose mmol/L	5.10 ± 0.43	5.41 ± 0.88	4.98 ± 1.13	NS
AspAt U/L	26.13 ± 5.08	32.71 ± 16.75	36.00 ± 4.85	0.048 *
ALT U/L	38.75 ± 20.71	76.86 ± 61.65	52.00 ± 23.00	NS
Aldosteron pg/mL	97.70 ± 53.77	145.36 ± 135.00	135.02 ± 138.60	NS

SDMA: Symmetric Dimethylarginine, Cyst C: Cystatin C, Mg: magnesium, Na: sodium, K: potassium, Cl: chlorine, Ca^2+^: ionized calcium, Fe: iron, CRP: C-reactive protein, AspAT: aspartate transaminase, ALT: alanine transaminase. * Statistically significant differences between the control group and kidney group, # statistically significant differences between the heart and the kidney group, ^ statistically significant differences between the healthy and the heart group, NS: statistically nonsignificant.

**Table 4:** Urinary examination of healthy dogs (control group), dogs with mitral regurgitation ACVIM Class Cc (heart group) and the kidney group (azotemic dogs). Average ± standard deviation.

Variable	Control Group	Heart Group	Kidney Group	*p* Value
Specific gravity	1.0371 ± 0.0099	1.0226 ± 0.0124	1.0118 ± 0.0051	0.007 *
pH	6.37 ± 0.44	6.30 ± 0.68	6.08 ± 0.66	NS
UPC	0.112 ± 0.089	0.490 ± 0.567	0.888 ± 0.783	NS
Urine albumin mg/L	4.125 ± 5.69	37.24 ± 36.20	75.83 ± 67.63	0.030 *
UAC mg/dL	1.76 ± 2.62	59.79 ± 71.35	201.91 ± 232.44	0.001 *^
Urine creatinine mmol/L	9734.5 ± 8982.82	8842.9 ± 5473.1	4416.7 ± 1343.8	<0.001 ^*
Podocytes	0	0.4 ± 0.5	0.3 ± 0.5	

UPC: Urine/Creatinine Ratio, UAC: Urine Albumin/Creatinine Ratio, UPoC: Urine Podocine/Creatinine Ratio. * Statistically significant differences between the control group and the kidney group, ^ statistically significant differences between the control and the heart group, NSS: not statistically significant.

**Table 5:** Echocardiographic parameters in the group of healthy dogs (control group), in the group of dogs with mitral regurgitation ACVIM Class Cc (heart group), and the kidney group (azotemic dogs). Average ± standard deviation.

	Control Group	HEART GROUP	Kidney Group	*p* Value
LA/Ao	1.369 ± 0.051	2.071 ± 0.535	1.329 ± 0.208	0.001 *#^
LVIDd mm	30.78 ± 6.08	38.42 ± 8.44	33.50 ± 5.89	NS
LVIDs mm	17.29 ± 7.23	22.45 ± 9.16	19.93 ± 2.76	NS
MR (+)	0.0 ± 0.0	2.7 ± 0.6	0.0 ± 0.0	<0.001 ^#
HR min ^−1^	131.4 ± 30.0	133.7 ± 26.7	135.2 ± 8.2	NS

LA/Ao: left atrium-to-aorta ratio, LVIDd: left ventricular end-diastolic diameter, LVIDs: left ventricular end-systolic diameter, FS: fractional shortening of left ventricle, MR: mitral regurgitation, HR: heart rate, SAP: systolic arterial pressure. * Statistically significant differences between the control group and the kidney group, # statistically significant differences between the heart and the kidney group, ^ statistically significant differences between the healthy and the heart group, NS: statistically nonsignificant.

**Table 6:** The Spearman correlation coefficient (r_s_) for podocin in each group: Healthy dogs (control group), group of dogs with ACVIM Class Cc mitral regurgitation (heart group), and kidney the group (azotemic dogs).

		Heart Group	Kidney Group
Podocin	Urea mmol/L:	NS	NS
	Urine creatinine mmol/L	NS	r_s_ = 0.828, *p* = 0.042 *
	Urine protein g/L	r_s_ = −0.558, *p* = 0.047 *	NS
	Cyst C mg/L:	NS	r_s_ = −0.840, *p* = 0.036 *

* *p* < 0.05, NS—statistically nonsignificant.
